# The association between body mass index and body bulk with externalizing behavior: a longitudinal twin study

**DOI:** 10.3389/frcha.2026.1905375

**Published:** 2026-08-04

**Authors:** Sofi Oskarsson, Adrian Raine, Anneli Andersson, Shichun Ling, Bridget Bertoldi, Laura A. Baker, Catherine Tuvblad

**Affiliations:** 1 School of Behavioural, Social and Legal Sciences, Örebro University, Örebro, Sweden; 2 Departments of Criminology, Psychiatry, and Psychology, University of Pennsylvania, Philadelphia, PA, United States; 3 School of Criminal Justice and Criminalistics, California State University - Los Angeles, Los Angeles, CA, United States; 4 Worcester Recovery Center and Hospital, Worcester, MA, United States; 5 Department of Psychology, University of Southern California, Los Angeles, CA, United States

**Keywords:** body bulk, body mass index, externalizing behavior, longitudinal design, twins

## Abstract

**Introduction:**

The current study examined longitudinal links between adolescent body mass index and body bulk with externalizing behaviors using data from the University of Southern California's Risk Factors for Antisocial Behavior twin project.

**Methods:**

The sample included 1,260 individuals (51% females), with 603 twin pairs (254 monozygotic; 349 dizygotic). Body mass index and body bulk were derived from measures of height and weight obtained at age 13-14. Externalizing behavior was measured using the Youth Self-Report at age 13-14 and the Adult Self-Report at age 19-20.

**Results:**

Higher body mass index and greater body bulk were associated with higher levels of externalizing behavior both concurrently at age 13-14, and prospectively at age 19-20 (*B*'s = 0.11-0.42, *p*'s < .05). Twin modeling indicated high heritability for body mass index and body bulk (h^2^ = 0.73-0.81) and moderate heritability for externalizing behavior (h^2^ = 0.31-0.41). Bivariate models suggested that genetic factors contributed to the modest covariance between body size and externalizing behavior, although the genetic correlation estimates were imprecise.

**Conclusion:**

Findings are consistent with the possibility that some genetic influences are shared between body size and externalizing behavior; however, the modest phenotypic associations and uncertainty surrounding the genetic correlation estimates warrant cautious interpretation.

## Introduction

Externalizing behavior is increasingly recognized as a public health concern, as it constitutes a major risk factor for adverse educational, social, psychiatric, and criminal outcomes (1–3). Externalizing behaviors typically emerge early in childhood, increase through adolescence and decline during adulthood for most individuals, although a smaller subgroup exhibits persistent high levels across the life course (1). Externalizing behavior refers to behavioral problems expressed outwardly through actions that negatively impact the external environment, such as aggression, rule-breaking, and delinquency (4, 5). Researchers have long sought to identify correlates of externalizing behavior to better understand their origins and develop strategies for prevention. Among these correlates, physical characteristics such as body size have been explored based on the notion that greater physical capacity may facilitate or reinforce aggressive or rule-breaking behavior (6, 7). Contemporary research has provided empirical support for this idea, showing positive associations between body mass index (BMI) and body bulk with externalizing outcomes (8, 9, 58). Building on this work, the present study aims to clarify these associations by examining the links between adolescent BMI and body bulk with externalizing behavior over time, while disentangling the genetic and environmental factors that may contribute to this overlap.

Body size can be characterized in different ways. The most commonly used measure is BMI, which reflects body mass relative to height (10) and is widely used as an indicator of overweight and obesity (37). However, BMI does not distinguish between individuals who have similar BMI values but differ substantially in their overall physical build or muscularity. An alternative measure is body bulk, which reflects overall physical size by combining information on height and weight (7). Whereas BMI primarily indexes relative adiposity at the population level, body bulk may better capture larger physical stature or formidability emphasized in evolutionary accounts. Thus, BMI and body bulk may show different associations with externalizing behavior.

### Evolutionary theory

Evolutionary perspectives propose that externalizing behavior (e.g., aggression, rule-breaking) may have been adaptive under certain ecological and social conditions by facilitating access to resources, social status, or mating opportunities (11–13). Because larger individuals are generally perceived as more physically formidable and dominant (14, 15), body size may alter the costs and benefits of externalizing behaviors. This framework therefore provides one theoretical account of why body size and externalizing behavior may be associated.

#### Empirical evidence on the association between body size and externalizing behavior

Several studies have reported positive associations between body size and externalizing behavior across childhood, adolescence, and adulthood. Importantly, most evidence in this area comes from observational studies and thus cannot distinguish causal effects from alternative explanations, including reversed causation, shared genetic liability, or environmental confounding. Despite these limitations, several longitudinal studies have reported positive associations between body size and later externalizing behavior, including aggression, rule-breaking, delinquency and violent offending, often measured through the Achenbach system or official records.

In a longitudinal cohort from Mauritius, greater body bulk at age three predicted aggression at age eleven (7), while another study found that higher body mass at age twelve was associated with increased aggression one year later (16). BMI has also been associated with more severe externalizing outcomes. For example, BMI at 12 months predicted an increased risk of violent offending from adolescence onward in a Finnish cohort (17), and a Swedish population-based study reported that each one-standard-deviation increase in BMI at age 18 was associated with a 16% higher risk of later violent criminal conviction (8). In adolescence and adulthood, taller and bulkier males have also been found to report greater involvement in physical fights (18). Consistent with these findings, a meta-analysis of 23 studies (*N* = 255,377) found that overweight and obese children and adolescents displayed higher levels of physical aggression than their normal-weight or underweight peers (*d* = 0.27), although substantial heterogeneity across studies was noted (19).

Research has also examined body size in relation to antisocial personality disorder, which represents one severe manifestation of the broader externalizing spectrum (20). One study reported that males meeting criteria for antisocial personality disorder were significantly taller and exhibited greater overall body bulk than controls, although BMI was not associated with the disorder (21). This finding raises the possibility that overall physical size rather than BMI alone, may be more closely related to some externalizing phenotypes. However, findings have not been entirely consistent. Another study observed a positive association between BMI and antisocial personality features among women but not men (22), while others reported that individuals with athletic physiques had higher rates of incarceration for violent offenses than those with other body types (23).

Not all studies have supported a positive association. One study found no significant association between BMI and externalizing behavior when distinguishing between persistent and adolescent-limited developmental trajectories, although the estimated odds ratios were generally above 1 and accompanied by wide confidence intervals (24). Similarly, others reported that shorter stature, rather than larger body size, was associated with violent convictions; however, this association was substantially attenuated after adjustment for intelligence, other physical characteristics, and childhood demographic factors, suggesting that confounding may partly account for the observed relationship (25).

Evidence also suggests that the association may operate in the opposite direction. One study found that externalizing behavior predicted later overweight, whereas overweight did not predict subsequent externalizing behavior (26). Likewise, others reported that behavioral problems during childhood and adolescence were associated with higher BMI in young adulthood (27). Alternatively, body size and externalizing behavior may share common risk factors, including socioeconomic disadvantage, temperamental characteristics, and self-regulatory difficulties, resulting in an association that does not reflect a direct causal relationship (26). Taken together, these findings suggest that the mechanisms linking body size and externalizing behavior remain uncertain.

#### Genetic contributions to externalizing behavior and body size

One possible explanation for reported associations between body size and externalizing behavior is that the two traits share common genetic influences. Both traits are known to be substantially heritable, raising the possibility that their phenotypic association reflects overlapping genetic liability rather than a direct causal relationship. Twin and family studies estimate that approximately half of the variation in externalizing behavior can be attributed to genetic factors (28–31), with estimates ranging from ∼30% to ∼60% depending on phenotypic definition. Body size is similarly influenced by genetic variation, with heritability estimates of around 30% for normal weight, 40%–50% for obesity, and as high as 60%–80% for severe obesity (32). This gradient has been corroborated in other studies, indicating that the heritability of BMI is not uniform but changes continuously across the BMI distribution, with higher heritability observed toward the upper end (33). While the heritable bases of externalizing behavior and BMI are well established, less is known about the extent to which their association is explained by shared genetic influences. Because genetic correlations are estimated from the covariance between traits, detecting shared genetic influences is challenging when phenotypic associations are small and generally requires relatively large samples. Clarifying the extent of shared genetic influences could improve our understanding of whether higher BMI confers increased risk for externalizing behavior, or whether both traits primarily reflect overlapping genetic liability.

#### The present study

The present study utilized a longitudinal twin cohort to examine whether body size in adolescence (ages 13–14 years), operationalized as BMI and body bulk, was associated with externalizing behavior, both concurrently and prospectively in early adulthood (ages 19–20 years). We further examined the genetic and environmental contributions to body size and externalizing behavior separately, and the extent to which their covariance reflected shared genetic and/or environmental influences. This approach allowed us to evaluate not only whether adolescent body size predicted later externalizing behavior, but also additional insights into the etiological processes underlying their association.

Based on earlier findings, we hypothesized the following:

1. Based on extensive evidence of a positive association between body size and externalizing behavior (19), we expected that our measures of body size would predict externalizing behavior. Specifically, we hypothesized that both BMI (16) and body bulk (7) would be positively associated with externalizing behavior, both concurrently and prospectively.
2. We expected that genetic factors would account for a substantial proportion of the variance in body size (32), while explaining less than half of the variance in externalizing behavior, consistent with prior estimates (30, 31).
3. Although prior research has not directly examined the genetic correlation between body size and externalizing behavior, evolutionary theory suggests a positive association. Larger body size may confer advantages in status competition and resource acquisition (13, 14), and externalizing behavior has been conceptualized as an adaptive strategy in such contexts (11). Given that both traits are heritable (30, 32), we hypothesized that common genetic factors would contribute to their association, both concurrently and prospectively.

## Methods

The present study builds on data from the University of Southern California's (USC) Risk Factors for Antisocial Behavior (RFAB) project, a prospective study of twins and triplets followed every two to three years beginning at ages 9–10 years and over five consecutive Waves (34). The RFAB study focuses on the development of externalizing behavior and its associated biological and social risk factors. Ethical approval was obtained from the Institutional Review Board at the USC (34).

### Participants

The RFAB sample includes twins and triplets who are ethnically and socioeconomically representative of the greater Los Angeles area, with approximately equal numbers of males and females. The study includes same-sex monozygotic (MZ) and dizygotic (DZ) pairs, as well as opposite-sex DZ pairs. Zygosity for same-sex pairs was primarily determined using DNA microsatellite analysis, which assesses concordance of DNA markers to identify identical genomes (35). In cases where DNA results were inconclusive, zygosity was assessed using the Twin Similarity Questionnaire (36), which has shown 90% concordance with DNA-based determination (35). Retention was high: by Wave 4, 529 of the original 614 families (86%) had returned for at least one follow-up assessment.

For the current study, the base sample consisted of 1,573 individuals (1,546 twins and 27 triplets). From these individuals, we excluded those who had missing data on all predictor variables (*n* = 24) and both outcome variables (*n* = 289). These exclusions resulted in an overall sample of 1,260 individuals (51% females). For twin analyses, we excluded all singletons and triplets after removing missing data (*n* = 52), making sure all twins had their co-twin and excluded those with missing data on zygosity (*n* = 2). This exclusion resulted in a twin sample of 1,206 unique individuals (51% females), equivalent to 603 twin pairs. Out of these twin pairs, 254 were MZ twins and 349 were DZ twins. Included and excluded participants did not differ significantly with respect to sex, age at first wave of data collection, or BMI at baseline (*p*'s > .05).

### Measures

#### Predictors

##### Body size

Height and weight were measured in the laboratory at USC at age 13–14 for 881 participants, while 325 individuals provided self-reported values. BMI was calculated as weight in kilograms divided by height in meters squared (kg/m^2^). Because the BMI distribution was positively skewed, BMI values were square-root transformed prior to analysis. BMI is often used as an indicator of obesity (37), yet individuals with the same BMI can vary markedly in overall body size. We therefore also derived a measure of body bulk following earlier work (7). Body bulk was calculated as the product of height (cm) and weight (kg), standardized (z-transformed), and shifted by adding a constant of 1. This constant shifts the distribution so that values are predominantly positive and does not affect the relative ordering of individuals or the associations with other variables. Higher scores indicate greater body bulk. BMI and body bulk were strongly correlated in the present sample (*r* = .83).

#### Outcome

##### Wave 3

At age 13–14, participants completed the Youth Self-Report (YSR) from the Achenbach System of Empirically Based Assessment [ASEBA (38)]. For the present study, we focused on the externalizing subscale, which consists of 30 items assessing delinquency and aggression (*α* = 0.86). Out of the current sample of 1,206 individuals, 731 individuals had available YSR-data at age 13–14. Example items include: “I hang around with kids who get in trouble”, “I tease others a lot”, and “I have a hot temper”. The YSR externalizing subscale has demonstrated strong validity for assessing problem behaviors in children and adolescents (39, 40).

##### Wave 5

At age 19–20, participants completed the Adult Self-Report (ASR), also from the ASEBA (41). For the present study, we used the externalizing subscale, which includes 29 items assessing aggressive and rule-breaking behaviors (*α* = 0.88). Out of the current sample of 1,206 individuals, 651 individuals had available YSR-data at age 19–20. Example items include: “I argue a lot”, “I am mean to others,” and “I break rules at work or elsewhere.” The ASR has demonstrated high reliability and validity in measuring adult problem behaviors (42).

Consistent with the ASEBA guidelines, each item on the YSR and the ASR was scored on a 3-point scale (0 = not true; 1 = somewhat true; 2 = very true/often true) and summed to produce a continuous externalizing score.

#### Covariates

Sex was included as a covariate given that the rates of externalizing behavior are consistently higher among males than females, a pattern observed across time, cultures, and multiple data sources (43–46).

### Statistical analysis

Data management and analysis were performed using R Studio version 2025.9.0.387 (47). First, zero-order correlations between body size measures and externalizing behavior were examined. Next, linear regressions models were used to estimate associations and 95% confidence intervals (CIs) between predictors and outcomes. In the first model, each predictor (BMI or body bulk) was entered with each outcome (i.e., externalizing behavior at age 13–14 or externalizing behavior at age 19–20) to obtain unadjusted estimates. In the second model, sex was included as a covariate. This resulted in two models per predictor-outcome combination. All regression models accounted for the non-independence of twin observations using a cluster robust sandwich estimator.

Subsequently, we used classical twin modeling in OpenMx (R) to estimate the contribution of genetic and environmental factors to the association between body size and externalizing behavior (48). Models were estimated using Full Information Maximum Likelihood (FIML), allowing all available observations to contribute to parameter estimation under the assumption that data were missing at random. These models decompose variance and covariance into additive genetic (A), shared environmental (C), and nonshared environmental (E) components by leveraging differences in genetic and environmental sharing between MZ and DZ twins. Both same-sex and opposite-sex DZ twin pairs were included in the analyses, with common genetic and environmental parameters estimated across males and females. Bivariate models were used to estimate genetic and environmental correlations between phenotypes, providing insight into the extent to which etiological factors are shared across traits (49). These analyses rely on key assumptions: MZ twins share 100% of their genetic make-up, DZ twins share, on average, 50% of their genes, shared environmental influences are assumed to affect MZ ad DZ twins equally, and nonshared environmental influences are uncorrelated and include measurement error.

#### Sensitivity analyses

To assess the robustness of our findings, we repeated all analyses using a subsample of participants whose height and weight were measured directly in the laboratory (*N* = 881) rather than obtained through self-report.

## Results

The mean (*M*) BMI at age 13–14 was 22.1 with a standard deviation (*SD*) of 4.8. Mean externalizing behavior scores were 10.4 (*SD* = 7.1) at ages 13–14 and 9.7 (*SD* = 8.1) at ages 19–20. Zero-order correlations in the full cohort and separately for MZ and DZ twins are presented in Table 1. At ages 13–14, BMI and body bulk were weakly, positively correlated with externalizing behavior both concurrently [*r*'s = 0.14, 95% CI: [0.07, 0.20]; *r*'s = 0.08, 95% CI: [0.02, 0.15]] and prospectively [*r*'s = 0.15, 95% CI: [0.08, 0.22]; *r*'s = 0.14, 95% CI: [0.06, 0.20]]. Intraclass correlations among DZ twins were about half the size of the intraclass correlations for MZ on all variables, indicating the importance of genetic influences on both body size and externalizing behavior.

**Table 1:** Phenotypic correlations and intraclass correlations for body mass index, bulk, and externalizing behavior at ages 13–14 and 19–20.

No of individuals	Full cohort	MZ	DZ
	1,206	508	698
Phenotypic *r* (95% CI) BMI age 13–14/EXT age 13–14	0.14 (0.07, 0.20)	—	—
Phenotypic *r* (95% CI) Bulk age 13–14/EXT age 13–14	0.08 (0.02, 0.15)	—	—
Phenotypic *r* (95% CI) BMI age 13–14/EXT age 19–20	0.15 (0.08, 0.22)	—	—
Phenotypic *r* (95% CI) Bulk age 13–14/EXT age 19–20	0.14 (0.06, 0.20)	—	—
ICC (95% CI) BMI age 13–14	—	0.89 (0.86, 0.92)	0.55 (0.46, 0.63)
ICC (95% CI) Bulk age 13–14	—	0.92 (0.89, 0.94)	0.52 (0.43, 0.61)
ICC (95% CI) EXT age 13–14	—	0.52 (0.40, 0.62)	0.32 (0.20, 0.43)
ICC (95% CI) EXT age 19–20	—	0.45 (0.33, 0.56)	0.19 (0.07, 0.30)

MZ, monozygotic; DZ, dizyotic; CI, confidence interval; BMI, body mass index; EXT, externalizing behavior; ICC, intraclass correlation.

### Associations between body size and externalizing behavior

Crude regression estimates are reported in the Supplementary materials (Table S1). Table 2 presents sex-adjusted regression models examining the associations between body size and externalizing behavior. At age 13–14, both higher BMI and greater body bulk were associated with higher externalizing behavior [*B* = 0.34, 95% CI: [0.18, 0.49], *p* < .001; *B* = 0.11, 95% CI: [0.02, 0.20], *p* < .05]. At age 19–20, BMI and body bulk remained positively associated with externalizing behavior [*B* = 0.42, 95% CI: [0.22, 0.61], *p* < .001; *B* = 0.19, 95% CI: [0.08, 0.30], *p* < .01].

**Table 2:** Linear regressions between BMI and Bulk as predictors with externalizing behavior as the outcome.

Outcome	Predictor	Estimate^a^	SE	95% CI	*p*-value
EXT age 13–14	BMI	0.34	0.08	0.18, 0.49	<.001
	Bulk	0.11	0.05	0.02, 0.20	.022
EXT age 19–20	BMI	0.42	0.10	0.22, 0.61	<.001
	Bulk	0.19	0.06	0.08, 0.30	.001

a Adjusted for sex.

EXT, externalizing behavior; BMI, body mass index; CI, confidence interval.

### Genetic and environmental contributions to body size and externalizing behavior

Table 3 shows the proportion of variance in BMI, body bulk, and externalizing behavior attributable to additive genetic (*h*^2^), shared environmental (*c*^2^), and unique environmental (*e*^2^) factors based on univariate ACE models. Additive genetic factors accounted for a substantial proportion of variance in body size, with BMI (*h*^2^ = 0.73) and body bulk (*h*^2^ = 0.81) at age 13–14 showing high heritability, while shared environmental influences were minimal and non-significant, and unique environmental effects were modest. In contrast, externalizing behavior was less strongly influenced by genetics at age 13–14 (*h*^2^ = 0.31) and at age 19–20 (*h*^2^ = 0.41). Unique environmental factors explained the largest proportion of variance in externalizing behavior (*e*^2^ ≈ 0.50), whereas shared environmental contributions were modest and non-significant both at age 13–14 and at age 19–20.

**Table 3:** Univariate model fit: proportion of variance in traits attributed to additive genetic (A) and unique environmental (E) factors based on ACE models.

Phenotype	*h* ^2^	*c* ^2^	*e* ^2^
BMI age 13–14	0.73 (0.59, 0.90)	0.17 (0.01, 0.32)	0.10 (0.08, 0.12)
Bulk age 13–14	0.81 (0.66, 0.93)	0.11 (0.00, 0.26)	0.08 (0.07, 0.10)
Ext age 13–14	0.31 (0.05, 0.57)	0.20 (0.00, 0.40)	0.49 (0.41, 0.59)
Ext age 19–20	0.41 (0.11, 0.54)	0.05 (0.00, 0.29)	0.54 (0.45, 0.65)

*h*^2^ = proportion of additive genetic factors; *c*^2^ = proportion of shared.

environmental factors; *e*^2^ = proportion of unique environmental factors.

Bivariate twin models were used to examine the genetic and unique environmental contributions to the phenotypic associations between body size and externalizing behavior (Table 4). Across both concurrent and prospective analyses, model estimates indicated that genetic factors accounted for a substantial proportion of the observed covariance, with unique environmental factors accounting for the remainder. For example, genetic influences accounted for 87% of the association between BMI at age 13–14 and concurrent externalizing behavior, while genetic contributions across analyses ranged from 77%–93%. However, it is important to note that the underlying phenotypic associations were modest in magnitude (*r* = .08–.15), indicating that the absolute magnitude of shared genetic influences was also small. Furthermore, confidence intervals around the genetic correlation estimate between body bulk at age 13–14 and externalizing behavior at age 13–14 included zero, indicating uncertainty regarding the strength of genetic overlap. Shared environmental contributions were fixed to zero because estimates for C were unstable and occasionally implausible. Likelihood-ratio tests comparing ACE and AE models indicated that dropping the shared environment did not significantly worsen model fit (*p*'s > .05), supporting the use of an AE model (see Supplementary Table S2 for ACE model estimates). Overall, these findings are consistent with the possibility that shared genetic factors contribute to the modest association between body size and externalizing behavior, although the magnitude of this overlap appears small and should be interpreted cautiously.

**Table 4:** Bivariate model estimates: genetic and environmental contributions to the phenotypic correlation between body size and externalizing behavior.

Phenotype pair	rA	rC^a^	rE	h^2^r	c^2^r^a^	e^2^r
BMI age 13–14 Ext age 13–14	0.18 (0.07,0.29)	NA	0.09 (−0.04, 0.22)	87%	NA	13%
Bulk age 13–14 Ext age 13–14	0.09 (−0.03, 0.20)	NA	0.08 (−0.05, 0.22)	80%	NA	20%
BMI age 13–14 Ext age 19–20	0.17 (0.04, 0.30)	NA	0.15 (0.00, 0.28)	77%	NA	23%
Bulk age 13–14 Ext age 19–20	0.18 (0.05, 0.30)	NA	0.04 (−0.11, 0.18)	93%	NA	7%

rA = genetic correlation; rE = unique environment correlation; h^2^r = proportion of correlation explained by genetic factors; e^2^r = proportion of correlation explained by unique environmental factors.

a Estimates for the shared environment component (C) was unstable across models for all traits, occasionally producing implausible values. Therefore, the C paths were fixed to 0, resulting in an AE model that yielded stable estimates of additive genetic (A) and unique environmental (E) variance components. Likelihood-ratio tests comparing the ACE and AE models indicated that dropping C did not significantly worsen model fit in any of the models tested (*p*'s > .05), supporting the AE model.

### Sensitivity analyses

Repeating analyses in a subsample of participants for whom data on height and weight was obtained in-lab rather than through self-report did not yield any differences in results. Results from these analyses show the same patterns as the analyses from the full sample (Supplementary Table S3-S6).

## Discussion

In the present study, we examined the association between adolescent body size and externalizing behavior, both concurrently (age 13–14) and prospectively (age 19–20), using a longitudinal twin cohort. Our findings add to the body of research showing that higher BMI and greater body bulk were modestly but consistently associated with higher externalizing behavior at both time points (19). Twin analyses indicated that body size was highly heritable, whereas externalizing behavior showed moderate heritability and substantial influence from individual-specific environmental factors. Bivariate models suggested that genetic factors contributed to the modest covariance between body size and externalizing behavior. These findings are consistent with the possibility of some shared genetic liability, although the small phenotypic associations and uncertainty surrounding the genetic correlation estimates mean that the extent of this overlap remains unclear.

Additionally, it is important to emphasize that the current study's findings should not be interpreted as indicating that individuals with larger body size are inherently predisposed toward antisocial, delinquent, or criminal behavior. Body size is associated with a wide range of social and health experiences, such as weight-based victimization (50), bullying (51), stigma (52), and adverse mental health outcomes (53), that may also influence behavioral development. Moreover, molecular genetic studies have documented positive genetic correlations between individual externalizing-related traits (e.g., ADHD, alcohol dependence, heavy smoking) and adiposity measures (54), consistent with shared disinhibition as a pleiotropic pathway. The observed genetic covariation in the present study may, at least in part, reflect this pathway rather than mechanisms specific to physical formidability. Likewise, the modest association observed are compatible with alternative explanations, including reverse causal pathways whereby externalizing tendencies contribute to later increases in body size through unhealthy lifestyle behaviors, as well as shared environmental influences.

Nevertheless, findings from the present study contribute to the substantial literature reporting a positive association between body size and externalizing behavior, as summarized in a meta-analysis (19). Our results are broadly consistent with previous cross-sectional (21) and longitudinal studies (7, 17) reporting positive associations between body size predicts and later externalizing outcomes. At the same time, it is important to acknowledge that not all studies have demonstrated this link. For example, earlier work reported null associations between BMI and antisocial behavior measured through self-report and official criminal records, though these null findings may reflect limited statistical power rather than the true absence of effect (24). Similarly, others found no association between height and weight and violent criminal convictions in a Swedish total-population sample after controlling for multiple confounders (25). In contrast, other studies using Swedish population-based registers have reported a positive association between BMI and violent criminal convictions (8), highlighting that the predictive value of body size may depend on how body composition is measured.

Our results further show that body bulk did not demonstrate stronger associations with externalizing behavior than BMI. Although previous work has suggested that body bulk may be more closely linked to antisocial tendencies than BMI, potentially because it reflects overall physical size or muscularity (21), our findings provided little support for this proposition. Rather, the associations between body size and externalizing behavior were broadly similar across measures, and BMI tended to emerge as the somewhat stronger predictor. One possible interpretation is that the aspects of physique relevant to externalizing behavior are already adequately captured by BMI, despite its limitations as a proxy for body composition. Alternatively, body bulk may not fully capture dimensions of physical formidability that are theoretically relevant to externalizing tendencies, such as muscularity, strength, or athletic capacity. Future research incorporating more direct measures of physical formidability may help clarify whether specific aspects of physique show stronger associations with externalizing behavior than general indicators of body size.

Our twin modeling results are consistent with previous research reporting substantial heritability for body size (32), although our estimates were at the upper end of those typically reported. One possible explanation is that the heritability of BMI varies across the BMI distribution, with higher estimates generally observed among individuals with obesity (33). In addition, sampling variability may have contributed to the relatively high estimates observed in the present study. For externalizing behavior, our heritability estimates ranged from 31%–41%, which aligns well with prior meta-analyses and twin studies (30, 31). To our knowledge, this is the first study to examine genetic correlations between body size and externalizing behavior both concurrently and longitudinally. Across models, the fitted twin models attributed a substantial proportion of the observed covariance to additive genetic factors. However, because the phenotypic associations were small and confidence intervals around several genetic correlation estimates were relatively wide, these findings should be interpreted cautiously.

From an evolutionary perspective, the finding that genetic factors accounted for most of the observed association between body size and externalizing behavior is broadly consistent with hypotheses proposing that physical formidability and some forms of externalizing behavior may have been linked over evolutionary history (11, 13, 19). At the same time, the present findings are equally compatible with other explanations. For example, the observed genetic overlap may reflect pleitropic genetic influences on traits such as impulsivity, reward sensitivity, or self-regulation, which are themselves associated with both body size and externalizing behavior (55–57). Because the present study cannot distinguish between these competing explanations, it does not provide direct evidence for evolutionary mechanisms. Rather, the findings should be interpreted as compatible with, but not confirming, evolutionary accounts of the association between body size and externalizing behavior.

Our findings should be considered in light of several limitations. First, externalizing behavior was assessed through self-report, and body size measures relied partly on self-reported height and weight. Although commonly used, self-reports may be influenced by recall or reporting biases. Future studies would benefit from incorporating multiple informants and objective measures to strengthen validity. Second, participants were drawn from the RFAB twin cohort in Southern California. While the sample is ethnically and socioeconomically diverse and broadly representative of the region (34, 35), results may not generalize to other cultural or geographic contexts. Third, our prospective analyses spanned from early adolescence (age 13–14) to early adulthood (age 19–20), and it remains uncertain whether similar associations extend into later adulthood. Fourth, although the longitudinal design allowed prospective analyses, it does not establish causality, and the observed associations may reflect reverse causal pathways or residual confounding by unmeasured environmental factors. Consequently, the observed phenotypic associations may partly reflect confounding by factors such as pubertal development, socioeconomic circumstances, or parenting practices. Future studies should examine the robustness of these associations after accounting for these and other potential confounders. Fifth, the twin models estimated common genetic and environmental parameters across males and females. Consequently, the present study was not designed to evaluate potential sex differences in the genetic and environmental influences underlying the association between body size and externalizing behavior. Future studies with larger samples could investigate this possibility. Finally, although associations between body size and externalizing behavior were statistically significant, effect sizes were small, which may limit their practical or clinical significance. Moreover, because genetic correlations are estimated from the covariance between traits, the modest phenotypic associations also limited the precision with which shared genetic influences could be estimated. Although the sample was relatively large for a longitudinal twin study, it may nevertheless have provided limited statistical power to detect modest genetic correlations. Accordingly, the genetic correlations should be viewed as preliminary and require replication in larger genetically informative samples.

## Conclusion

The present study suggest that body size and externalizing behavior are modestly associated both concurrently and prospectively during adolescence and early adulthood. Twin modeling suggested that additive genetic factors contributed to a substantial proportion of the observed covariance, although the modest phenotypic associations and limited power to estimate genetic correlations warrant cautious interpretation. These findings are consistent with the possibility that some of the same genetic factors contribute to variation in both body size and externalizing behavior. Future research should build on these results by examining gene-environment interplay, extending analyses to diverse populations and developmental periods, exploring these relationships within families and investigating whether specific dispositional traits mediate or moderate the association between body size and externalizing behavior. Overall, the findings contribute to a broader understanding of the factors that may underlie the association between physical and behavioral characteristics across development.

## Data Availability

The data analyzed in this study is subject to the following licenses/restrictions: The datasets generated and/or analyzed during the current study are not publicly available. Requests to access these datasets should be directed to lbaker@usc.edu.
