# Supplementary material for: The association between body mass index and body bulk with externalizing behavior: a longitudinal twin study
Source: Front Child Adolesc Psychiatry. 2026 Aug 4;5:1905375. doi: 10.3389/frcha.2026.1905375 (PMC13481729; doi:10.3389/frcha.2026.1905375)
Supplement: Supplementary file 1 [file Table1.docx]

Supplementary material

**Table S1.** Crude estimates from linear regressions between BMI and Bulk as predictors with Externalizing behavior as the outcome.

**Table S2.** Model fit statistics for ACE and AE bivariate twin models examining associations between body size (BMI and bulk) and externalizing behavior.

**Table S3.** Phenotypic correlations and intraclass correlations in a subsample of in-lab participants for body mass index, bulk, and externalizing behavior at ages 13-14 and 19-20

**Table S4.** Linear regressions between BMI and Bulk as predictors with Externalizing behavior as the outcome in a subsample of in-lab participants

**Table S5.** Univariate model fit: Proportion of variance in traits attributed to additive

genetic (A) and unique environmental (E) factors based on ACE models in a subsample of in-lab participants

**Table S6.** Bivariate model fit: Genetic and unique environmental contributions to the phenotypic correlation between traits

**Table S1**

*Crude estimates from linear regressions between BMI and Bulk as predictors with Externalizing behavior as the outcome*

| **Outcome** | **Predictor** | **Estimate^a^** | **SE** | **95% CI** | ***p*-value** |
| --- | --- | --- | --- | --- | --- |
| EXT Wave 3^b^ | BMI | 0.32 | 0.08 | 0.17, 0.48 | <.001 |
|  | Bulk | 0.10 | 0.04 | 0.02, 0.19 | .016 |
| EXT Wave 5^c^ | BMI | 0.42 | 0.10 | 0.22, 0.62 | <.001 |
|  | Bulk | 0.20 | 0.05 | 0.10, 0.31 | <.001 |

Abbreviations: EXT = Externalizing behavior; BMI = body mass index; CI = confidence interval

^b^ Ages 13-14 years

^c^ Ages 19-20 years

**Table S2**

*Model fit statistics for ACE and AE bivariate twin models examining associations between body size (BMI and bulk) and externalizing behavior.*

| Phenotype pair | ACE -2LL | ACE df | ACE AIC | AE -2LL | AE df | AE AIC | Δχ² | Δdf | p |
| --- | --- | --- | --- | --- | --- | --- | --- | --- | --- |
| BMI age 13-14  Ext age 13-14 | 4040.70 | 1957 | 4062.70 | 4047.91 | 1960 | 4063.91 | 7.21 | 3 | .066 |
| Bulk age 13-14  Ext age 13-14 | 5000.34 | 1957 | 5022.34 | 5004.67 | 1960 | 5020.67 | 4.32 | 3 | .228 |
| BMI age 13-14  Ext age 19-20 | 4139.38 | 1877 | 4161.38 | 4144.16 | 1880 | 4160.16 | 4.78 | 3 | .189 |
| Bulk age 13-14  Ext age 19-20 | 5094.70 | 1877 | 5116.70 | 5097.03 | 1880 | 5113.03 | 2.33 | 3 | .507 |

Note. ACE = additive genetic (A), shared environmental (C), and non-shared environmental (E) model; AE = additive genetic and non-shared environmental model. -2LL = -2 log-likelihood; df = degrees of freedom; AIC = Akaike Information Criterion; Δχ² = likelihood-ratio chi-square test comparing the AE model with the ACE model; Δdf = difference in degrees of freedom. Lower AIC values indicate better relative model fit. Non-significant likelihood-ratio tests indicate that constraining the shared environmental component (C) to zero did not significantly worsen model fit, supporting the AE model.

**Table S3**

*Phenotypic correlations and intraclass correlations for body mass index, bulk, and externalizing behavior at ages 13-14 and 19-20 in a subsample of in-lab participants*

|  | **Full cohort** | **MZ** | **DZ** |
| --- | --- | --- | --- |
| No of individuals | 881 | 362 | 516 |
| Phenotypic *r* (95% CI) BMI age 13-14/EXT age 13-14 | 0.13 (0.07, 0.20) | - | - |
| Phenotypic *r* (95% CI) Bulk age 13-14/EXT age 13-14 | 0.08 (0.02, 0.15) | - | - |
| Phenotypic *r* (95% CI) BMI age 13-14/EXT age 19-20 | 0.14 (0.07, 0.21) | - | - |
| Phenotypic *r* (95% CI) Bulk age 13-14/EXT age 19-20 | 0.13 (0.06, 0.21) | - | - |
| ICC (95% CI) BMI age 13-14 | - | 0.89 (0.85, 0.92) | 0.54 (0.46, 0.63) |
| ICC (95% CI) Bulk age 13-14 | - | 0.91 (0.88, 0.93) | 0.51 (0.43, 0.61) |
| ICC (95% CI) EXT age 13-14 | - | 0.52 (0.40, 0.62) | 0.33 (0.21, 0.44) |
| ICC (95% CI) EXT age 19-20 | - | 0.46 (0.32, 0.58) | 0.18 (0.04, 0.32) |

Abbreviations: MZ = monozygotic; DZ = dizyotic; CI = confidence interval; BMI = body mass index; EXT = Externalizing behavior; ICC = intraclass correlation

**Table S4**

*Linear regressions between BMI and Bulk as predictors with Externalizing behavior as the outcome in a subsample of in-lab participants*

| **Outcome** | **Predictor** | **Estimate^a^** | **SE** | **95% CI** | ***p*-value** |
| --- | --- | --- | --- | --- | --- |
| EXT age 13-14 | BMI | 0.31 | 0.08 | 0.15, 0.46 | <.001 |
|  | Bulk | 0.11 | 0.05 | 0.02, 0.20 | .017 |
| EXT age 19-20 | BMI | 0.39 | 0.10 | 0.19, 0.59 | <.001 |
|  | Bulk | 0.19 | 0.06 | 0.08, 0.30 | .001 |

^a^ Adjusted for sex

Abbreviations: EXT = Externalizing behavior; BMI = body mass index; CI = confidence interval

**Table S5**

*Univariate model fit: Proportion of variance in traits attributed to additive*

*genetic (A) and unique environmental (E) factors based on ACE models in a*

*subsample of in-lab participants*

|  | h^2^ | c^2^ | e^2^ |
| --- | --- | --- | --- |
| BMI age 13-14 | 0.74 (0.59, 0.91) | 0.17 (0.00, 0.31) | 0.10 (0.08, 0.12) |
| Bulk age 13-14 | 0.84 (0.68, 0.93) | 0.07 (0.00, 0.24) | 0.09 (0.07, 0.11) |
| Ext age 13-14 | 0.33 (0.05, 0.59) | 0.18 (0.00, 0.41) | 0.49 (0.40, 0.61) |
| Ext age 19-20 | 0.41 (0.16, 0.53) | 0.02 (0.00, 0.21) | 0.57 (0.47, 0.69) |

Note: h^2^ = proportion of additive genetic factors; c^2^ = proportion of shared

environmental factors; e^2^ = proportion of unique environmental factors.

**Table S6**

*Bivariate model fit: Genetic and unique environmental contributions to the phenotypic correlation between traits in a subsample of in-lab participants*

|  | rA | rC^a^ | rE | h^2^r | c^2^r^a^ | e^2^r |
| --- | --- | --- | --- | --- | --- | --- |
| BMI age 13-14 Ext age 13-14 | 0.16 (0.04,0.26) | NA | 0.12 (-0.02, 0.26) | 81% | NA | 19% |
| Bulk age 13-14 Ext age 13-14 | 0.09 (-0.02, 0.20) | NA | 0.09 (-0.05, 0.24) | 77% | NA | 23% |
| BMI age 13-14 Ext age 19-20 | 0.16 (0.02, 0.29) | NA | 0.14 (-0.02, 0.28) | 75% | NA | 25% |
| Bulk age 13-14 Ext age 19-20 | 0.18 (0.04, 0.31) | NA | 0.03 (-0.12, 0.19) | 93% | NA | 7 % |

Note: rA = genetic correlation; rE = unique environment correlation; h^2^r = proportion of correlation explained by genetic factors; e^2^r = proportion of correlation explained by unique environmental factors

^a^ Estimates for the shared environment component (C) was unstable across models for all traits, occasionally producing implausible values. Therefore, the C paths were fixed to 0, resulting in an AE model that yielded stable estimates of additive genetic (A) and unique environmental (E) variance components. Likelihood-ratio tests comparing the ACE and AE models indicated that dropping C did not significantly worsen model fit in any of the models tested (p’s > .05), supporting the AE model.
